# The Level of Health Culture Related to Heart Disease Among Students of the Faculty of Artificial Intelligence at Al-Balqa Applied University

**DOI:** 10.7759/cureus.39832

**Published:** 2023-06-01

**Authors:** Bandar Ghazal

**Affiliations:** 1 Faculty of Medicine, Al-Balqa Applied University, Al-Salt, JOR

**Keywords:** awareness, university student, continuing health education, heart disease, health culture

## Abstract

Increasing awareness of any disease, especially heart disease, is crucial to improve health culture in general. The lack of communication between the different departments of social and health institutions may hinder such increasing awareness due to the lack of enough research that highlights this problem. As health culture education related to heart diseases raises young people’s awareness, it improves their lives by developing their knowledge and changing their attitudes, habits, and behaviours towards the risk factors related to such diseases. Therefore, this research aimed to determine the level of health culture related to heart disease among students at Al-Balqa Applied University. The descriptive approach was used in its analytical and survey styles to achieve the research objective, and the research sample consisted of 221 male and female students. The results showed that the total score for the level of health culture related to heart disease among the students was average. In light of the results, the researcher presented several recommendations. The most important are holding health education and awareness seminars and workshops for university students in the field of heart disease and its prevention and activating the role of Al-Balqa Applied University in the continuous guidance and counselling of students in all disciplines and levels to increase the health culture of heart disease.

## Introduction

Many diseases have spread in the society in our time and are closely related to life’s nature, pace, and various tensions, in addition to food’s quality and food consumption pattern. Perhaps the most prominent diseases are diabetes, hypertension, obesity, and heart diseases, which have spread in our societies to sound the alarm [1]. Therefore, heart disease and stroke constitute a burden on citizens and governments in developing countries [2].

Several risk factors related to the development of heart diseases as mostly known are aging, family history of heart disease, stress, smoking, heavy alcohol consumption, unhealthy dietary habits (high in fat and carbohydrates and low in fibers) and lack of exercise [1]. In fact, the increased incidence of obesity, hypertension, hyperlipidemia, stroke and smoking among younger people (ages 35-64) are putting them at risk for heart disease earlier in life. Therefore it is becoming more common to observe young individuals in their 40s and 50s suffering from a heart disease [3]. The World Health Organization confirms that 17.9 million people die yearly due to cardiovascular diseases. According to the World Heart Federation, the death rate from cardiovascular diseases is about (33%), i.e. nineteen million deaths every year [4]. Thus, by spreading health culture, the necessary precautions can be taken to prevent and combat heart diseases.

University students are considered among society’s most important age groups because this stage is the country’s economic and political future, therefore, as they become future parents their level of health culture will provide their children with an incubator of healthy behaviors, which can be a protective dose against diseases [5]. Taking this into consideration, health culture has become one of the primary goals in most educational institutions, especially universities since scientific and health developments are progressing in a manner characterized by acceleration and successive rhythm than ever before [6].

As health culture education related to heart diseases raises young people’s awareness, it improves their lives by developing their knowledge and changing their attitudes, habits, and behaviors towards the risk factors mentioned earlier [7]. As an academic institution, one of the university’s duties is to educate students on everyday habits and to solve students' problems, including low health culture and awareness, by providing them with appropriate healthy behavioral patterns and developing these behaviors [8]. Universities should also conduct many programs and activities aimed at raising the level of health culture and awareness among their students [9]. Considering that the university, in its contemporary meaning, is an institution that has its own specificity and features, distinguishing it from other societal institutions, it occupies a distinguished position in change and development, the most important features of the modern era to face challenges and keep up with developments [10-11].

Increasing awareness on any disease especially heart diseases is crucial to improve health culture in general. The lack of communication between the different departments of social and health institutions may hinder such increasing such awareness due to the lack of enough research that highlights this problem. Therefore, this research aimed to determine the level of health culture related to heart disease among students at Al-Balqa Applied University.

## Materials and methods

This research is a descriptive cross-sectional study, with analytical and survey methods, to achieve its objectives. The research tool was distributed to 511 students at the Faculty of Artificial Intelligence at Al-Balqa University students in the academic year 2022-2023. Only 252 questionnaires were returned back, and 31 were excluded because they were not filled up properly to be able to execute the proper analysis. Therefore, the remaining 221 samples were eventually included in the study. All participants consented to take part in the current research. This research was conducted in accordance with the Declarations of Helsinki and after verbal approval from the Deanship of Scientific Research and Innovation at the university.

The research tool (Table 1) was prepared after referring to the theoretical literature and literature review on the subject. The tool consisted of 25 items representing behavioral practices related to health education about heart disease. The answer scale consisted of five answers: Strongly agree (5), agree (4), neutral (3), disagree (2), and strongly disagree (1). All items were formulated to express a positive meaning because it reflects the level of culture on heart disease health.

## Results

Table 1 shows that the level of health culture related to heart disease among students at Al-Balqa University was "average" in general, with an arithmetic mean±SD of 2.94±0.687 and a percentage of 58.95%. Table 1 also shows that item (6) got the first rank, which states: “I eat fresh fruits and vegetables more than canned ones”, with an arithmetic mean of 4.03, a percentage of 80.63%, and a “high” approval level, followed by item (16) in the second rank, which states: “I refrain from smoking regular or electronic cigarettes and avoid places where others smoke” with an arithmetic mean of 3.95, a percentage of 79.09%, and a “high” approval level. Item (4) came in the third rank, which states: “I refrain from eating food fortified with processed carbohydrates”, with an arithmetic mean of 3.54, a percentage of 70.95%, and a “high” approval level.

It also appears from the table that item (12) got the last rank, which states: “I discuss with specialists in aspects related to public health and heart health in particular” with an arithmetic mean of 1.59 and a percentage of 31.94% and the level of approval "very low". It appears from Table 1 that the following eight items have a “high” level of health culture, with arithmetic means that ranged between 3.46 and 4.03 and a percentage that ranged between 69.23 and 80.63%:

• I eat fresh fruits and vegetables more than canned ones.

• I refrain from smoking regular or electronic cigarettes and avoid places where others smoke.

• I refrain from eating food fortified with processed carbohydrates.

• I get enough sleep, not less than (8) hours a day, and I avoid staying up late for a long time.

• I refrain from taking drugs and alcohol.

• I don’t chew tobacco or smoke pipes or hookahs. 

• I see a doctor when symptoms appear (heart palpitations, nausea, indigestion, heartburn, stomach pain, shortness of breath, prolonged cough, swelling of the feet and ankles, etc.).

• I conduct periodic medical examinations to detect hidden diseases.

In contrast, the following eight items obtained an “average” health culture level, with arithmetic means that ranged between 2.77 and 3.30 and a percentage that ranged between 55.56 and 66.15%, which are:

• I read the instructions (leaflet) about calories when buying foodstuffs.

• I make sure to buy low-fat or skimmed dairy products.

• I avoid using stimulants and sports stimulants or drinks that contain caffeine in large quantities.

• I follow scientific health publications and inform those around me (family, university, community) of any accurate health information.

• I deal with stress through physical activities, relaxation exercises, or meditation.

• I conduct a periodic and complete medical examination at least every year.

• I commit to a blood test (cholesterol) every six months at least.

• I eat lean meat and fish.

Finally, eight items obtained a “low” level of health culture, with arithmetic means that ranged between 2 and 2.60 and a percentage that ranged between 40 and 52.03%, which are:

• I eat foods that contain healthy fats, such as olive oil or vegetable oils.

• I avoid having a lot of sweets, chocolate, and soft drinks.

• I maintain an appropriate weight and avoid gaining or losing weight.

• I eat a variety of appropriate meals daily and at specific times.

• I avoid accidents, behaviors, and exercises that are risky for my heart and health.

• I keep a careful personal and family health history, especially with regard to heart disease.

• I carefully read the instructions written on the medicines and follow them when buying medicines.

• I follow a diet low in salt and saturated and trans fats.

**Table 1:** The arithmetic means, standard deviations, and percentages of the items of the tool

M	Item	Arithmetic mean	Standard deviation	Percentage	Item rank	Approval level
6	I eat fresh fruits and vegetables more than canned ones	4.03	1.001	80.63	1	high
16	I refrain from smoking regular or electronic cigarettes and avoid places where others smoke	3.95	1.012	79.09	2	high
4	I refrain from eating foods fortified with processed carbohydrates	3.54	0.816	70.95	3	high
9	I get enough sleep, not less than 8 hours a day, and I avoid staying up late for a long time	3.52	1.463	70.49	4	high
3	I avoid drugs and alcohol	3.48	0.501	69.77	5	high
17	I don’t chew tobacco or smoke a pipe or a hookah	3.48	0.518	69.68	6	high
8	I see a doctor when symptoms appear (heart palpitations, nausea, indigestion, heartburn, stomach pain, shortness of breath, prolonged cough, swelling of the feet and ankles, etc.)	3.46	0.591	69.23	7	high
20	I conduct periodic medical examinations to detect hidden diseases	3.46	0.543	69.23	8	high
7	I read the instructions (leaflet) related to calories when purchasing foodstuffs	3.30	0.795	66.15	9	Average
21	I make sure to buy low-fat or fat-free dairy products	3.09	0.983	61.9	10	Average
11	I avoid stimulants, sports stimulants, or drinks that contain caffeine in large quantities	3.04	0.998	60.90	11	Average
10	I follow scientific health publications and inform those around me (family, university, community) of any accurate health information	3.02	0.176	60.45	12	Average
24	I deal with stress through physical activities, relaxation exercises or meditation	3.00	0.190	60.18	13	Average
13	I do a periodic and complete medical examination at least every year	2.99	0.261	59.91	14	Average
14	I commit to having my blood (cholesterol) checked at least every six months	2.98	0.986	59.63	15	Average
22	I eat lean meat and fish	2.77	0.416	55.56	16	Average
23	I eat foods that contain healthy fats, such as olive oil or vegetable oils	2.60	0.599	52.03	17	low
5	I avoid having a lot of sweets, chocolate, and soft drinks	2.52	0.543	50.58	18	low
1	I maintain an appropriate weight and avoid gaining or losing weight	2.52	0.509	50.49	19	low
2	I eat a variety of appropriate meals daily and at specific times	2.49	0.510	49.86	20	low
19	I avoid accidents, behaviors, and exercises that are risky for my heart and health	2.34	0.530	46.87	21	low
15	I keep a careful personal and family health history, especially regarding heart diseases	2.25	0.503	45.06	22	low
18	I carefully read the instructions written on the medicines and follow them when buying medicines	2.15	1.067	43.07	23	low
25	I follow a diet low in salt and saturated and trans fats.	2	1.022	40.00	24	low
12	I discuss with specialists the aspects related to public health and heart health, in particular	1.59	0.636	31.94	25	very low
Total		2.94	0.687	58.95	-	Average

To ensure the construct validity of the research tool, it was presented to ten arbitrators and specialists to ensure its validity and language and propose appropriate amendments. Moreover, to ensure the validity of the internal consistency of the tool, the Pearson correlation coefficient was calculated to find out the correlation of each item, as shown in Table 2. It is clear from Table 2 that the correlation coefficient values for the tool items ranged between 0.806 and 0.599. These values are appropriate and indicate the tool’s validity and suitability for what it was developed for.

**Table 2:** Correlation coefficients for the items of the tool

Item No	Correlation coefficient	Significance level	Item No	Correlation coefficient	Significance level
1	0.631*0	0.0000	14	0.710*0	0.0000
2	0.693**0	0.0000	15	0.662**0	0.0000
3	0.599**0	0.0000	16	0.697**0	0.0000
4	0.688**0	0.0000	17	0.648**0	0.0000
5	0.754**0	0.0000	18	0.685**0	0.0000
6	0.712*0	0.0000	19	0.699*0	0.0000
7	0.806**0	0.0000	20	0.683**0	0.0000
8	0.769**0	0.0000	21	0.751**0	0.0000
9	0.744**0	0.0000	22	0.656**0	0.0000
10	0.677**0	0.0000	23	0.649**0	0.0000
11	0.721**0	0.0000	24	0.648**0	0.0000
12	0.688**0	0.0000	25	0.677**0	0.0000
13	0.773**0	0.0000			

The stability coefficient of the tool was calculated using the Cronbach-alpha equation, as shown in Table 3. It appears from Table 3 that the value of the stability coefficient for the tool was 0.856, which is considered suitable for this research and a clear indication of the stability and validity of the tool.

**Table 3:** Stability coefficient of the tool

	The number of items	Stability coefficient
Total	25	0.856

## Discussion

The current study discusses the level of health culture related to awareness of heart diseases among students of the Faculty of Artificial Intelligence at Al-Balqa Applied University, Jordan. Taking into consideration the risk factors leading to heart problems, the results reveal that the student's level of health culture related to heart disease is generally considered "average". They have a high level of obtaining information through the internet and social media but poor awareness of the importance of reading medical brochures and seeking for medical examinations. These results cover the health culture levels related to heart diseases which are also mentioned in other studies [12-16] and indicate a decline in health culture related to risk factors leading to heart disease development. This can be explained by the students’ habituation to fast food and sweets and lack of interest in their weight, as well as the lack of sports centers and free playgrounds for useful exercises, games, and sports activities.

The level of health culture awareness of heart disease among our university students is considered average and is similar to other university students in the region [14] and [16]. However, this result differs from a study by Al-Hudaybi [12], which showed that the level of health awareness among students was low, and Al-Arjan [13] which found that the level of health awareness was high.

A study from a Jordanian University in another city (Mutah University) showed that the level of health awareness among their students was considered high [13], whereas it was average among students at a different campus of our university (Zarqa University College at Al-Balqa Applied University) [14]. Therefore, the researchers recommended intensifying our university courses in health culture and raising awareness of the importance of health awareness in its various fields [14]. Another study showed that the level of awareness towards governmental health initiatives differed among university students in other countries, for example, the level of health awareness was 40% among students at Punjabi University, Patiala, India, and 60% among students at the University of East in Bangladesh [17]. The largest percentage of students at Tabuk University had a high level of health culture in light of their National Vision 2030 [7]. Since the internet is an important source for obtaining health information, several studies showed that the students' general health information at different universities was influenced by internet, media, social media, and visual media, for example, at Tabuk University [7] and Al-Balqa Applied University [15].

## Conclusions

In conclusion and in light of the results of this research, it is recommended to hold health education and awareness seminars and workshops for university students on heart disease and its prevention and to include topics related to heart disease and health in university curricula. Also, it is recommended to activate the role of the university’s media in health culture and the continuous guidance and counseling of students in all disciplines and levels of study to increase the health culture of heart disease. In addition, conducting various sports activities in the university and encouraging students to participate, and providing health services and consultations to university students regarding healthy behaviors helps prevent heart disease. Finally, it is important to carry out similar studies in health culture with other diseases.
